# Comparative effects of microvascular and macrovascular disease on the risk of major outcomes in patients with type 2 diabetes

**DOI:** 10.1186/s12933-017-0574-y

**Published:** 2017-07-27

**Authors:** Kamel Mohammedi, Mark Woodward, Michel Marre, Stephen Colagiuri, Mark Cooper, Stephen Harrap, Giuseppe Mancia, Neil Poulter, Bryan Williams, Sophia Zoungas, John Chalmers

**Affiliations:** 1 The George Institute for Global Health, University of Sydney, Sydney, NSW Australia; 2 INSERM, UMRS 1138, Centre de Recherche des Cordeliers, Paris, France; 3 Department of Diabetology, Endocrinology and Nutrition, Assistance Publique Hôpitaux de Paris, Bichat Hospital, DHU FIRE, Paris, France; 4 The George Institute for Global Health, University of Oxford, Oxford, UK; 5 Department of Epidemiology, Johns Hopkins Bloomberg School of Public Health, Johns Hopkins University, Baltimore, MD USA; 6 University Paris Diderot, Sorbonne Paris Cité, UFR de Médecine, Paris, France; 7 Boden Institute of Obesity, Nutrition, Exercise & Eating Disorders, Sydney Medical School, University of Sydney, Sydney, NSW Australia; 8 Baker IDI Heart and Diabetes Institute, Melbourne, VIC Australia; 9 The University of Melbourne and Royal Melbourne Hospital, Melbourne, VIC Australia; 10 The University of Milan-Bicocca and Istituto Auxologico Italiano, Milan, Italy; 11 The International Centre for Circulatory Health, National Heart and Lung Institute, Imperial College, London, UK; 12 Institute of Cardiovascular Sciences, University College London (UCL) and NIHR UCL Hospitals Biomedical Research Centre, London, UK; 13 Monash Centre for Health Research and Implementation, School of Public Health and Preventive Medicine, Monash University, Clayton, VIC Australia

**Keywords:** Type 2 diabetes, Microvascular disease, Macrovascular disease, Mortality

## Abstract

**Background:**

Microvascular disease is associated with a high risk of macrovascular events in patients with type 2 diabetes, but the impact of macrovascular disease on the risk of microvascular events remains unknown. We sought to evaluate the respective effects of prior microvascular and macrovascular disease on the risk of major outcomes, including microvascular events, in these patients.

**Methods:**

Participants in the Action in Diabetes and Vascular Disease: PreterAx and DiamicroN Modified-Release Controlled Evaluation (ADVANCE) trial (n = 11,140) and the ADVANCE-ON post-trial study (n = 8494) were categorized into 4 groups at baseline: dual absence of microvascular or macrovascular disease (n = 6789), presence of microvascular disease alone (n = 761), macrovascular disease alone (n = 3196), and both (n = 394). Outcomes were all-cause mortality, major macrovascular events (MACE), and major clinical microvascular events.

**Results:**

All-cause mortality, MACE, and major clinical microvascular events occurred in 2265 (20%), 2166 (19%), and 807 (7%) participants respectively, during a median follow-up of 9.9 (inter-quartile interval 5.6–10.9) years. The adjusted hazard ratios [95% CI] of death, MACE, and major clinical microvascular events were each greater in patients with baseline microvascular disease (1.43 [1.20–1.71], 1.64 [1.37–1.97], and 4.74 [3.86–5.82], respectively), macrovascular disease (1.43 [1.30–1.57], 2.04 [1.86–2.25], and 1.26 [1.06–1.51]) or both (2.01 [1.65–2.45], 2.92 [2.40–3.55], and 6.30 [4.93–8.06]) compared with those without these conditions. No interaction was observed between baseline microvascular and macrovascular disease for these events. The addition of microvascular disease (change in c-statistic [95% CI] 0.005 [0.002–0.008], p = 0.02) or macrovascular disease (0.005 [0.002–0.007], p < 0.0001) considered separately or together (0.011 [0.007–0.014], p < 0.0001) improved the discrimination and the classification (integrated discrimination improvement (IDI): 0.013 [0.010–0.016], p < 0.001; net reclassification improvement (NRI): 0.021 [0.011–0.032], p < 0.001) of the risk of all-cause mortality. Microvascular disease improved discrimination (0.009 [0.003–0.014]) and classification (IDI: 0.008 [0.006–0.010]; NRI: 0.011 [0.001–0.020]) of MACE. Baseline macrovascular disease modestly enhanced IDI (0.002 [0.001–0.002]) and NRI (0.041 [0.002–0.087]), but not discrimination, of major clinical microvascular events.

**Conclusions:**

Microvascular and macrovascular disease are independently associated with the 10-year risk of death, MACE, and major clinical microvascular events in patients with type 2 diabetes. The coexistence of these conditions was associated with the highest risks.

**Electronic supplementary material:**

The online version of this article (doi:10.1186/s12933-017-0574-y) contains supplementary material, which is available to authorized users.

## Background

Type 2 diabetes is a leading cause of microvascular complications and confers an excess risk of cardiovascular disease and death [1, 2]. Microvascular and macrovascular complications often occur concomitantly, and share similar risk factors and pathological pathways [3–5]. The presence of microvascular disease increases the risk of cardiovascular morbidity and mortality in people with type 2 diabetes, independent of the major established cardiovascular risk factors [6]. However, the impact of macrovascular disease on the risk of microvascular events has not been fully investigated. We sought to evaluate the impact of microvascular and macrovascular disease, considered individually, and together, on the risk of death and major macrovascular and microvascular events in patients with type 2 diabetes participating in the Action in Diabetes and Vascular Disease: PreterAx and DiamicroN Modified-Release Controlled Evaluation (ADVANCE) trial (ClinicalTrials.gov Number, NCT00145925) and ADVANCE-ON post-trial study (ClinicalTrials.gov Number, NCT00949286).

## Materials and methods

### Participants

ADVANCE was a multi-national randomized trial testing the effect of intensive glucose control (using a gliclazide-MR) and routine blood pressure treatment (using a fixed-dose combination of perindopril and indapamide) on the risk of major microvascular and macrovascular events in 11,140 patients with type 2 diabetes and at least one other cardiovascular risk factor or pre-existing cardiovascular disease [7–9]. Subsequently, 8494 of the surviving participants were enrolled in the post-trial observational study (ADVANCE-ON). The design and characteristics of participants have been previously described [8–10]. The Institutional Ethics Committee of each participating centre approved the ADVANCE and ADVANCE-ON protocols, and all participants provided written informed consent.

### Definition of microvascular and macrovascular disease at baseline

At baseline, microvascular disease was defined as the presence of macroalbuminuria (urinary albumin to creatinine ratio (ACR) >300 mg/g), requirement of retinal photocoagulation therapy, proliferative retinopathy, macular oedema, or diabetes-related blindness. Macrovascular disease was defined as the presence, at baseline, of myocardial infarction, stroke, coronary artery bypass graft, percutaneous transluminal coronary angioplasty, hospital admission for unstable angina or transient ischaemic attack, lower-extremity amputation of at least one digit secondary to arterial insufficiency, or a peripheral revascularisation procedure. Participants were categorized into four baseline groups: absence of both microvascular and macrovascular disease, presence of microvascular disease alone, presence of macrovascular disease alone, and presence of both microvascular and macrovascular disease.

### Definition of outcomes

The primary outcomes were all-cause mortality, major macrovascular events (MACE: a composite of nonfatal myocardial infarction, nonfatal stroke, or cardiovascular death), and major clinical microvascular events (a composite of end-stage renal disease (ESRD), defined as requirement for renal-replacement therapy; death induced by renal disease; requirement for retinal photocoagulation; or diabetes-related blindness in either eye). The secondary outcomes were cardiovascular death, fatal or nonfatal myocardial infarction, fatal or nonfatal stroke, ESRD or renal death, and requirement for retinal photocoagulation or blindness. Outcomes were adjudicated by an independent End Point Adjudication Committee in the ADVANCE trial, through to the end of randomized allocation, and were reported by investigators without adjudication in the ADVANCE-ON study, in accordance with its pre-specified protocol [10].

### Statistical analyses

Continuous variables were summarized as mean (SD) or, for those with a skewed distribution, median (interquartile range). Categorical variables were summarized as the number of patients with corresponding percentage. Characteristics of participants according to status of microvascular and macrovascular disease at baseline were compared using Chi squared, ANOVA, or Kruskal–Wallis tests.

Cumulative incidence curves were used to plot survival (outcome-free) rates during follow-up, and compared using the log-rank test. Cox proportional hazards regression models were fitted to estimate hazard ratios (HRs), with associated 95% confidence intervals (CI), for outcomes by joint status of microvascular and macrovascular disease at baseline. Analyses were adjusted for randomized study allocations plus every potential confounding variable that was significantly different between microvascular and macrovascular disease at baseline: sex, age, region of origin (Asia: Philippines, China, Malaysia, and India; established market economies: Australia, Canada, France, Germany, Ireland, Italy, Netherlands, New Zealand, United Kingdom; and Eastern Europe: Czech Republic, Estonia, Hungary, Lithuania, Poland, Russia, Slovakia), body mass index, duration of diabetes, HbA1c, systolic blood pressure, antihypertensive treatment, estimated glomerular filtration rate (eGFR; computed by the Chronic Kidney Disease–Epidemiology Collaboration equation [11]) and its square, urinary ACR, LDL- and HDL-cholesterol, and history of ever smoking (basic model). The proportional hazards assumption was checked using the Schoenfeld residuals method.

We tested multiplicative interaction between baseline history of microvascular disease and macrovascular disease on the risk of each outcome by including these two individual variables and their product within Cox models.

Harrell’s c-statistic [12], net reclassification improvement (NRI) and integrated discrimination improvement (IDI) were used to compare discrimination and classification of primary outcomes, assessed using survival methodology, between two prognostic models: basic model and basic model plus baseline history of microvascular or macrovascular disease (as appropriate). Since we assessed the additive value of microvascular disease (including macroalbuminuria) on the risk of all-cause mortality and MACE, urinary ACR was not included in the basic model in these analyses. We also evaluated the individual and joint prognostic value of urinary ACR (as a continuous variable) and diabetic retinopathy, added to the basic model, on the discrimination of all-cause mortality and MACE.

Sensitivity analyses were conducted after including further components of microvascular disease at baseline: (i) chronic kidney disease (CKD defined as eGFR <60 ml/min/1.73 m^2^) or (ii) diabetic peripheral neuropathy (defined as a disturbance of 10-g monofilament sensation or absence of ankle reflex in both feet). We also evaluated the associations of microvascular and macrovascular disease at baseline on the risk of MACE and major clinical microvascular events after treating non-renal and non-cardiovascular death as a competing risk using the Fine and Gray method [13].

Statistical analyses were performed using SAS software, version 9.3 (SAS Institute, www.sas.com), and Stata software version 13 (StataCorp, www.stata.com). A P value less than 0.05 was considered significant.

## Results

### Clinical characteristics at baseline

Among 11,140 participants enrolled in ADVANCE trial, 761 (6.8%) had microvascular disease alone, 3196 (28.7%) had macrovascular disease alone, and 394 (3. 5%) had both at baseline (Additional file 1: Table S1). Patients with microvascular disease alone at baseline had a longer duration of diabetes and higher HbA1c, systolic blood pressure, and ACR levels, whereas those with macrovascular disease alone were more frequently men, from established market economies, ever smokers, and treated with antihypertensive, lipid lowering and antiplatelet drugs (Table 1).

**Table 1:** Clinical characteristics according to microvascular or macrovascular disease at baseline

	Overall (n = 11,140)	History of microvascular or macrovascular disease at baseline				
		Dual absence (n = 6789)	Microvascular alone (n = 761)	Macrovascular alone (n = 3196)	Both (n = 394)	p
Male sex, n (%)	6407 (57.5)	3628 (53.4)	404 (53.1)	2107 (65.9)	268 (68.0)	<0.0001
Region of origin: Asia, n (%)	4136 (37.1)	2525 (37.2)	350 (46.0)	1115 (34.9)	146 (37.1)	<0.0001
Region of origin: established market economies, n (%)	4862 (43.7)	3012 (44.4)	292 (38.4)	1389 (43.5)	169 (42.9)	
Region of origin: Eastern Europe, n (%)	2142 (19.2)	1252 (18.4)	119 (15.6)	692 (21.6)	79 (20.0)	
Age (years): mean (SD)	65.8 (6.4)	65.9 (6.3)	65.3 (6.4)	65.6 (6.6)	67.0 (6.6)	<0.0001
Duration of diabetes (years): mean (SD)	7.9 (6.4)	7.6 (6.1)	10.3 (7.3)	7.7 (6.3)	10.2 (7.2)	<0.0001
Body mass index (kg/m^2^): mean (SD)	28.3 (5.2)	28.4 (5.3)	27.7 (5.2)	28.4 (4.9)	28.2 (5.4)	0.002
Systolic blood pressure (mmHg): mean (SD)	145 (22)	145 (21)	149 (24)	144 (22)	148 (23)	<0.0001
Diastolic blood pressure (mmHg): mean (SD)	81 (11)	81 (11)	81 (12)	80 (11)	81 (11)	0.41
Use of antihypertensive treatment, n (%)	7655 (68.7)	4357 (64.2)	521 (68.5)	2466 (77.2)	311 (78.9)	<0.0001
HbA1c (%): mean (SD)	7.5 (1.6)	7.5 (1.6)	7.9 (1.7)	7.4 (1.5)	7.9 (1.6)	<0.0001
HbA1c (mmol/mol): mean (SD)	59 (17)	58 (17)	63 (19)	57 (16)	62 (18)	
eGFR (ml/min/1.73 m^2^)	74 (18)	76 (17)	73 (20)	73 (18)	69 (20)	<0.0001
Urinary ACR (mg/g): median (Q1, Q3)	15 (7, 40)	13 (7, 31)	49 (12, 390)	15 (7, 36)	86 (13, 461)	<0.0001
Serum total cholesterol (mmol/l): mean (SD)	5.2 (1.2)	5.3 (1.2)	5.3 (1.2)	5.0 (1.2)	4.9 (1.2)	<0.0001
Serum HDL cholesterol (mmol/l): mean (SD)	1.3 (0.4)	1.3 (0.4)	1.3 (0.4)	1.2 (0.3)	1.2 (0.3)	<0.0001
Serum LDL cholesterol (mmol/l): mean (SD)	3.1 (1.0)	3.2 (1.0)	3.2 (1.0)	3.0 (1.1)	2.9 (1.0)	<0.0001
Serum triglycerides (mmol/l): median (Q1, Q3)	1.6 (1.2, 2.3)	1.6 (1.2, 2.3)	1.6 (1.2, 2.3)	1.7 (1.2, 2.3)	1.6 (1.1, 2.2)	0.05
History of ever smoking, n (%)	4674 (42.0)	2702 (39.8)	271 (35.6)	1529 (47.8)	172 (43.7)	<0.0001

Comparison of qualitative and quantitative parameters were performed using Chi square and ANOVA tests, respectively. Kruskal–Wallis test was used for variables with skewed distribution (urinary albumin-creatinine ratio and triglycerides). p < 0.05 was significant

Established market economies: Australia, Canada, France, Germany, Ireland, Italy, Netherlands, New Zealand, United Kingdom; Eastern Europe: the Czech Republic, Estonia, Hungary, Lithuania, Poland, Russia, Slovakia; Asia: Philippines, China, Malaysia, India. eGFR, computed by the Chronic Kidney Disease Epidemiology Collaboration equation. ACR, Albumin to Creatinine Ratio. Use of lipid lowering drugs: statins or other hypolipidemic agents

### Incidence of major outcomes during follow-up according to status of microvascular and macrovascular disease at baseline

**Fig. 1:**
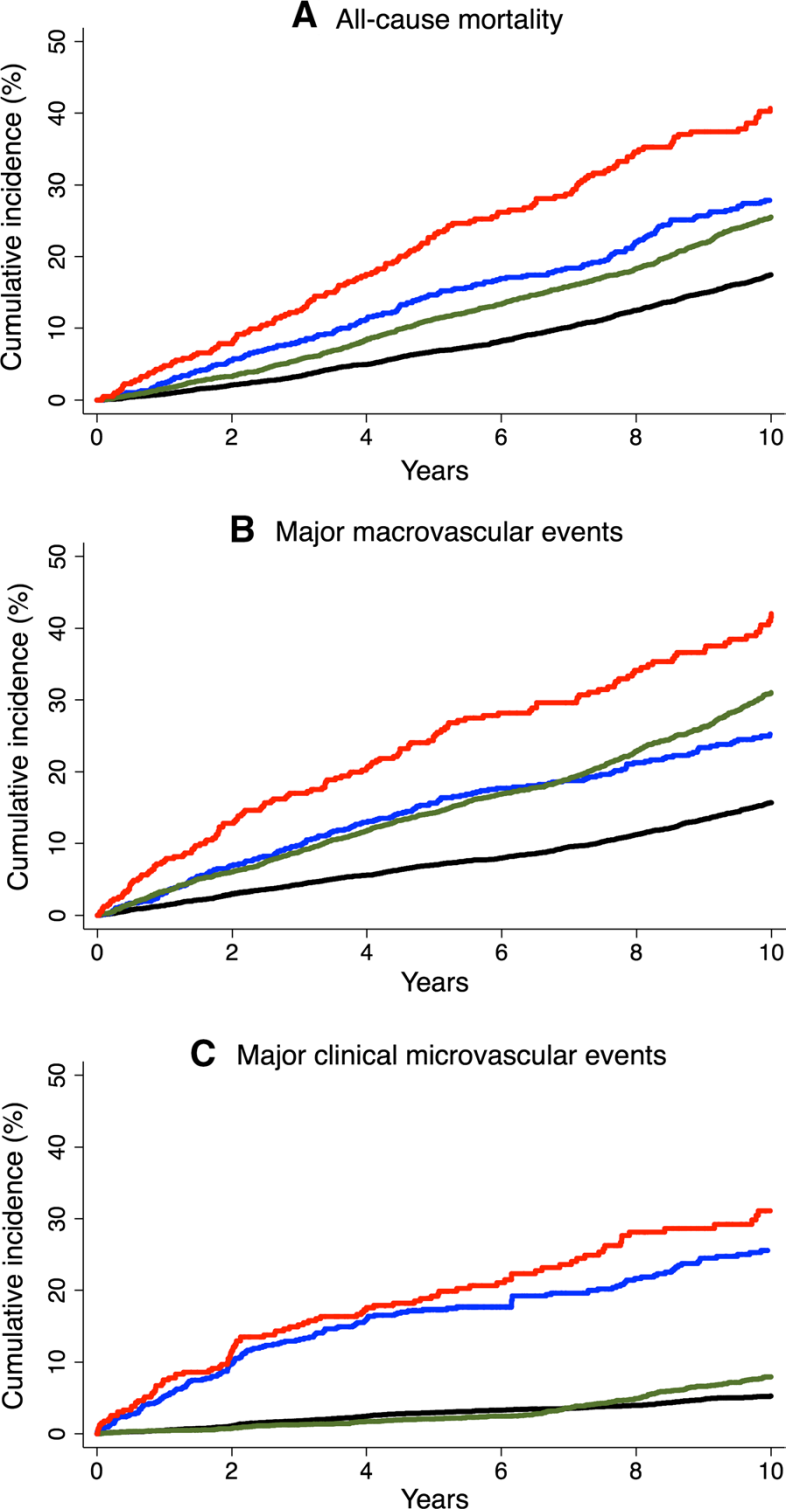
Cumulative incidence of outcomes during follow-up according to status of microvascular and macrovascular disease at baseline. **a** All-cause mortality. **b** Major macrovascular events. **c** Major clinical microvascular disease (p < 0.0001 for all). *Black line* absence of both macrovascular and microvascular disease. *Blue line* presence of microvascular disease alone. *Green line* presence of macrovascular disease alone. *Red line* presence of both microvascular and macrovascular disease

All-cause mortality, MACE, and major clinical microvascular events occurred in 2265 (20.3%), 2166 (19.4%), and 807 (7.2%) participants, respectively, during a median overall follow-up of 9.9 (inter-quartile interval 5.6–10.9) years. Their incidence rates were 2.4, 2.4, and 0.9 per 100 person-years, respectively. The risks of these respective outcomes were higher in patients with baseline history of only microvascular (HRs [95% CI] 1.43 [1.20–1.71], p < 0.0001; 1.64 [1.37–1.97], p < 0.0001; and 4.74 [3.86–5.82], < 0.0001) and of only macrovascular disease (1.43 [1.30–1.57], p < 0.0001; 2.04 [1.86–2.25], p < 0.0001; and 1.26 [1.06–1.51], p = 0.01), compared with patients without either of these conditions (Fig. 1; Table 2). The highest risks were observed in patients with both conditions at baseline (2.01 [1.65–2.45], p < 0.0001; 2.92 [2.40–3.55], p < 0.0001; and 6.30 [4.93–8.06], p < 0.0001). There was no evidence of interaction between microvascular and macrovascular disease at baseline on the risks of all-cause mortality (p = 0.89), MACE (p = 0.25), or major clinical microvascular events (p = 0.75).

**Table 2:** Distibution of patients, event rate (per 100 person years) and hazard ratios (HRs) for outcomes during follow-up, according to the history of microvascular or macrovascular disease at baseline

	History of microvascular or macrovascular disease Number of events (event rate)				Microvascular disease alone vs. dual absence		Macrovascular disease alone vs. dual absence		Both micro- and macrovascular disease vs. dual absence	
	Dual absence (n = 6789)	Microvascular alone (n = 761)	Macrovascular alone (n = 3196)	Both (n = 394)	HR (95% CI)	P	HR (95% CI)	P	HR (95% CI)	P
All-cause mortality	1136 (1.9)	201 (3.3)	773 (2.9)	155 (5.3)	1.43 (1.20 to 1.71)	<0.0001	1.43 (1.30 to 1.57)	<0.0001	2.01 (1.65 to 2.45)	<0.0001
Major macrovascular events	970 (1.7)	177 (3.1)	872 (3.6)	147 (5.6)	1.64 (1.37 to 1.97)	<0.0001	2.04 (1.86 to 2.25)	<0.0001	2.92 (2.40 to 3.55)	<0.0001
Cardiovascular death	396 (0.7)	98 (1.6)	406 (1.5)	88 (3.0)	1.96 (1.52 to 2.52)	<0.0001	2.13 (1.84 to 2.46)	<0.0001	3.19 (2.44 to 4.17)	<0.0001
Myocardial infarction	310 (0.5)	57 (1.0)	295 (1.1)	60 (2.2)	1.68 (1.22 to 2.30)	0.001	1.94 (1.65 to 2.29)	<0.0001	3.60 (2.63 to 4.92)	<0.0001
Stroke	446 (0.8)	67 (1.1)	402 (1.6)	53 (1.9)	1.32 (0.99 to 1.76)	0.06	2.15 (1.87 to 2.47)	<0.0001	2.36 (1.71 to 3.25)	<0.0001
Major clinical microvascular events	342 (0.6)	170 (3.2)	198 (0.8)	97 (3.9)	4.74 (3.86 to 5.82)	<0.0001	1.26 (1.06 to 1.51)	0.01	6.30 (4.93 to 8.06)	<0.0001
Retinal photocoagulation or blindness	284 (0.5)	143 (2.7)	166 (0.6)	76 (3.1)	5.28 (4.25 to 6.56)	<0.0001	1.34 (1.10 to 1.63)	0.003	6.98 (5.33 to 9.14)	<0.0001
ESRD or renal death	70 (0.1)	36 (0.6)	36 (0.1)	26 (0.9)	1.95 (1.12 to 3.37)	0.02	0.91 (0.60 to 1.38)	0.66	2.71 (1.53 to 4.81)	0.0006

Dual absence means absence of both macrovascular and microvascular disease at baseline. HRs estimated using Cox proportional hazards regression models, adjusting for sex, age, region of origin (established market economies, Eastern Europe and Asia), BMI, duration of diabetes, HbA1c, systolic blood pressure, antihypertensive treatment, eGFR and its square, urinary albumin-creatinine ratio (normoalbuminuria, microalbuminuria and macroalbuminuria), LDL- and HDL-cholesterol, history of ever smoking, and randomized study allocations

Comparable results were observed with secondary endpoints, except for the absence of association of baseline macrovascular disease with the risk of ESRD or renal death (Table 2).

### Additive values of microvascular or macrovascular disease at baseline in discrimination and classification of outcomes during follow-up

The addition of microvascular disease (change in c-statistic [95% CI] 0.005 [0.002–0.008], p = 0.02) or macrovascular disease (0.005 [0.002–0.007], p < 0.0001) considered separately or together (0.011 [0.007–0.014], p < 0.0001) improved the discrimination, as well as the classification (IDI: 0.013 [0.010–0.016], p < 0.001; NRI: 0.021 [0.011–0.032], p < 0.001) of the risk of all-cause mortality. The addition of ACR (0.010 [0.006–0.014], p < 0.0001) displayed a higher improvement of death discrimination than diabetic retinopathy (0.002 [0.001–0.004], p = 0.01).

The addition of baseline microvascular disease to established cardiovascular risk factors improved the discrimination (0.009 [0.003–0.014], p = 0.002) and classification (IDI: 0.008 [0.006–0.010], p < 0.001; NRI: 0.011 [0.001–0.020], p = 0.02) of MACE. The improvement in discrimination of MACE was similar after addition of either urinary ACR (0.008 [0.003–0.013], p = 0.002) or diabetic retinopathy (0.007 [0.002–0.011], p = 0.005). Adding both urinary ACR and diabetic retinopathy together yielded the highest increase in discrimination of MACE (0.014 [0.007–0.021], p < 0.0001).

Baseline macrovascular disease modestly enhanced IDI (0.002 [0.001–0.002]), p < 0.001 and NRI (0.041 [0.002–0.087], p < 0.0001), but not discrimination (c-statistic), of major clinical microvascular events (Table 3).

**Table 3:** Harrell’s c-statistics, NRI, IDI for risk of major outcomes according to traditional risk factors without and with history of microvascular or macrovascular disease at baseline

Risk of all-cause mortality		P
C-statistic (95% CI) for basic model	0.704 (0.693 to 0.716)	
Change in C-statistic (95% CI) for basic model + microvascular disease	0.005 (0.002 to 0.008)	0.02
Change in C-statistic (95% CI) for basic model + macrovascular disease	0.005 (0.002 to 0.007)	<0.0001
Change in C-statistic (95% CI) for basic model + microvascular disease + macrovascular disease	0.011 (0.007 to 0.014)	<0.0001
IDI (95% CI)	0.013 (0.010 to 0.016)	<0.001
Continuous NRI (95% CI)	0.275 (0.227 to 0.325)	<0.001
Categorical NRI (95% CI)	0.021 (0.011 to 0.032)	<0.001
*Risk of major macrovascular events (MACE)*		
C-statistic (95% CI) for basic model	0.648 (0.631 to 0.665)	
Change in C-statistic (95% CI) for basic model + microvascular disease	0.009 (0.003 to 0.014)	0.002
IDI (95% CI)	0.008 (0.006 to 0.010)	<0.001
Continuous NRI (95% CI)	0.120 (0.073 to 0.167)	<0.001
Categorical NRI (95% CI)	0.011 (0.001 to 0.020)	0.02
*Risk of major clinical microvascular events*		
C-statistic (95% CI) for basic model	0.664 (0.639 to 0.689)	
Change in C-statistic (95% CI) for basic model + macrovascular disease	0.004 (−0.003 to 0.011)	0.25
IDI (95% CI)	0.002 (0.001 to 0.002)	<0.001
Continuous NRI (95% CI)	0.211 (0.112 to 0.305)	<0.001
Categorical NRI (95% CI)	0.041 (0.002 to 0.087)	0.03

Basic model: sex, age, region of origin, BMI, duration of diabetes, HbA1c, systolic blood pressure, antihypertensive treatment, eGFR and its square, urinary albumin-creatinine ratio (for major clinical microvascular events), LDL- and HDL-cholesterol, history of ever smoking, and study allocations. IDI (integrated discrimination improvement) and NRI (net reclassification improvement) tests performed for basic model plus history of microvascular (risk of MACE), macrovascular disease (risk of major clinical microvascular events) or both (risk of all-cause mortality) at baseline, compared to basic model alone. Analyses performed in patients free of baseline history of microvascular disease (risk of major clinical microvascular events), or free of macrovascular disease (risk of MACE)

### Sensitivity analyses

Comparable associations were observed between baseline microvascular disease, including CKD or peripheral diabetic neuropathy, and the risk of outcomes, except for myocardial infarction or stroke (Additional file 1: Tables S2, S3). Baseline microvascular or macrovascular disease remained significantly associated with MACE and major clinical microvascular events when we corrected for competing risk of non-renal and non-cardiovascular death (Additional file 1: Table S4).

## Discussion

This study demonstrates the independent association of microvascular or macrovascular disease at baseline with excess risks of all-cause mortality, MACE, and major clinical microvascular events in patients with type 2 diabetes followed for a median duration of 9.9 years. The presence of both conditions led to the highest risks. Baseline microvascular disease enhanced discrimination and classification of MACE, while baseline macrovascular disease modestly improved classification, but not discrimination, of major clinical microvascular events. Overall, the improvement in discrimination and classification of outcomes was quiet modest, but statistically significant. Interestingly, urinary ACR and diabetic retinopathy yielded together the highest improvement of MACE supporting their additive value in the prediction of macrovascular disease. Of note, both ACR and retinopathy were independent components of the 10-year prognostic vascular risk score recently reported by our group in the ADVANCE-ON stud [14].

### Microvascular disease and risk of MACE

Our findings support previous studies showing that microvascular disease is an important predictor of future macrovascular disease and death [6, 15]. ADVANCE participants with microvascular disease alone at baseline displayed similar hazard ratios for all-cause mortality or MACE, compared to those with macrovascular disease alone, suggesting that microvascular impairment plays an important role in the development of diabetic angiopathy. A recent population-based cohort study has shown that retinal and skin microvascular abnormalities occurred early in prediabetes, were more severe in type 2 diabetes, and may contribute to the development of cardiovascular disease [16]. Other studies reported a high prevalence of coronary microvascular dysfunction in patients with type 2 diabetes free for known cardiovascular disease [17, 18].

### Macrovascular disease and risk of major clinical microvascular events

We evaluated, for the first time, the impact of macrovascular disease at baseline on the 9.9-year risk of major clinical microvascular events in patients with type 2 diabetes. Macrovascular disease at baseline was significantly associated with an elevated risk of the composite major microvascular endpoint, as well as retinal photocoagulation or blindness, but not ESRD or renal death. We recently reported that the history of peripheral arterial disease was associated with the risk of severe diabetic retinopathy, but not renal endpoints in ADVANCE and ADVANCE-ON studies [19]. Based on their poor prognosis, patients with chronic macrovascular disease at baseline may have died before experiencing ESRD during the follow-up. The impact of macrovasular disease on the risk of major clinical microvascular events seems to be weaker than the effect of microvascular disease on MACE risk, but there was no evidence of an interaction between the two conditions at baseline on the risk of any measured outcome. Despite their common background, these conditions had independent effects, suggesting that different mechanisms may be involved in the impact of microvascular and macrovascular disease on future vascular events. Hence there is a need to evaluate new predictors for both microvascular and macrovascular events. For instance, a high index of microcirculatory resistance and low coronary flow reserve were recently found associated with poor cardiovascular prognosis in patients with intermediate coronary stenosis (29% with type 2 diabetes) [20].

### Physiopathological mechanisms linking microvascular and macrovascular disease

Several pathways may explain the relationship between microvascular and macrovascular disease in patients with diabetes. Diabetic microvascular complications are mainly caused by prolonged exposure to high glucose levels. Diabetes is also associated with accelerated atherosclerosis affecting large vessels [21, 22]. Atherosclerosis is more prevalent in diabetic patients with microvascular disease compared to those without [21, 22]. However, it is still unknown why intensive glucose control does not yield the same benefit on macrovascular events as observed for microvascular outcomes [23]. Chronic hyperglycaemia causes vascular damage through activation of major biochemical paths including polyol pathway flux, increased formation of advanced glycation end products (AGEs), increased expression of AGEs receptor and its activating ligands, activation of protein kinase C isoforms, and overactivity of the hexosamine pathway [24]. Numerous lines of evidence suggest that these biochemical abnormalities may be activated by mitochondrial overproduction of reactive oxygen species (ROS) induced by hyperglycaemia [25]. The excess of ROS production with decreased in antioxidant capacity lead to oxidative stress, which plays an important role in the premature vascular morbidity and mortality in patients with diabetes [26]. Oxidative stress impairs endothelial function and endothelium-dependent vasodilation by inactivation of NO, and induces cell proliferation, hypertrophy, cardiac remodeling, apoptosis, and low-grade inflammation in endothelial and smooth cells of the vascular wall [27–30]. Moreover, oxidative stress is associated with many other accelerating conditions including insulin resistance, metabolic syndrome, hypertension, dyslipidaemia, and obesity, leading to both microvascular and macrovascular disease [25, 31]. The depletion of circulating stem cells may also explain the association between microvascular and macrovascular disease. A recent study has shown lower CD34^+^ and CD34^+^CD133^+^ cells in type 2 diabetic patients with cardiovascular events compared to those without [32]. The reduced levels of circulating progenitor cells improve the prediction of both macrovascular and microvascular events [32, 33]. The remodeling of bone marrow involves neurovascular changes and vascular abnormalities comparable to the known microangiopathy seen in the kidney and the retina [34].

### Strengths and limitations

The main strength of our study is the evaluation of the individual and the combined impact of microvascular and macrovascular disease at baseline on the risk of death and major microvascular and macrovascular outcomes in a large international cohort of patients with type 2 diabetes followed for a median duration of 9.9 years. The main limitation is the absence of biochemical renal assessment during the ADVANCE-ON follow-up, which may underestimate the association between baseline macrovascular disease and kidney disease. Furthermore, peripheral diabetic neuropathy was not investigated as an outcome in the post-trial, ADVANCE-ON, observational study. Although comparable findings were observed when we evaluated the associations of microvascular disease at baseline, including the history of peripheral diabetic neuropathy, with the main outcomes.

## Conclusion

The presence of microvascular or macrovascular disease at baseline is independently associated with increased risk of death, MACE, and major clinical microvascular events in people with type 2 diabetes followed for a median duration of 9.9 years, and their coexistence has an additive prognostic value. These findings encourage consideration of prior microvascular disease including retinal status, in addition to traditional cardiovascular risk factors, in selecting participants for forthcoming clinical trials aiming to assess vascular endpoints in patients with type 2 diabetes.

## Additional file

**Additional file 1: Table S1.** Components of microvascular and macrovascular disease at baseline. **Table S2.** Distibution of patients, event rate (per 100 person years) and hazard ratios (HRs) for outcomes during follow-up, according to the history of microvascular (including chronic kidney disease) or macrovascular disease at baseline. **Table S3.** Distibution of patients, event rate (per 100 person years) and hazard ratios (HRs) for outcomes during follow-up, according to the history of microvascular (including peripheral neuropathy) or macrovascular disease at baseline. **Table S4.** Subdistribution hazard ratios for outcomes during follow-up according to the history of microvascular or macrovascular disease at baseline with correction for competing risk of non-renal and non-cardiovascular death.

## Abbreviations

AGEs: advanced glycation end products
ACR: albumin to creatinine ratio
ADVANCE: Action in Diabetes and Vascular Disease: PreterAx and DiamicroN Modified-Release Controlled Evaluation trial
CKD: chronic kidney disease
CI: confidence interval
eGFR: estimated glomerular filtration rate
ESRD: end stage renal disease
HR: hazard ratio
IDI: integrated discrimination improvement
MACE: major macrovascular events
NRI: net reclassification improvement
ROS: reactive oxygen species
SD: standard deviation
