# Supplementary material for: Comparative effects of microvascular and macrovascular disease on the risk of major outcomes in patients with type 2 diabetes
Source: Cardiovasc Diabetol. 2017 Jul 27;16:95. doi: 10.1186/s12933-017-0574-y (PMC5530952; doi:10.1186/s12933-017-0574-y)
Supplement: Supplementary file 1 — Additional file 1: Table S1. Components of microvascular and macrovascular disease at baseline. Table S2. Distibution of patients, event rate (per 100 person years) and hazard ratios (HRs) for outcomes during follow-up, according to the history of microvascular (including chronic kidney disease) or macrovascular disease at baseline. Table S3. Distibution of patients, event rate (per 100 person years) and hazard ratios (HRs) for outcomes during follow-up, according to the history of microvascular (including peripheral neuropathy) or macrovascular disease at baseline. Table S4. Subdistribution hazard ratios for outcomes during follow-up according to the history of microvascular or macrovascular disease at baseline with correction for competing risk of non-renal and non-cardiovascular death. [file 12933_2017_574_MOESM1_ESM.docx]

**Additional Table S1. Components of microvascular and macrovascular disease at baseline**

|  | Microvascular disease alone (n=761) | Macrovascular disease alone (n=3196) | Both micro- and macrovascular disease (n=394) |
| --- | --- | --- | --- |
| Macroalbuminuria (urinary albumin to creatinine ratio > 300 mg/g) | 249 (62) | 0 | 155 (38) |
| Requirement of retinal photocoagulation therapy | 270 (67) | 0 | 134 (33) |
| Proliferative retinopathy | 269 (70.0) | 0 | 115 (30) |
| Macular oedema | 110 (64) | 0 | 63 (36) |
| Diabetes-related blindness in either eye | 63 (59) | 0 | 44 (41) |
| Myocardial infarction | 0 | 1183 (89) | 151 (11) |
| Stroke | 0 | 894 (87) | 129 (13) |
| Coronary artery bypass graft or percutaneous transluminal coronary angioplasty | 0 | 859 (90) | 92 (10) |
| Hospital admission for unstable angina | 0 | 1088 (90) | 126 (10) |
| Hospital admission for transient ischaemic attack | 0 | 476 (89) | 59 (11) |
| Lower-extremity amputation secondary to arterial insufficiency | 0 | 44 (68) | 21 (32) |
| Peripheral revascularisation procedure (surgery or angioplasty) | 0 | 185 (86) | 31 (14) |

Data expressed as number (percent).

**Additional Table S2. Distibution of patients, event rate (per 100 person years) and hazard ratios (HRs) for outcomes during follow-up, according to the history of microvascular (including chronic kidney disease) or macrovascular disease at baseline**

|  | History of microvascular or macrovascular disease  Number of events (event rate) | | | | Microvascular disease  alone vs. dual absence | | Macrovascular disease  alone vs. dual absence | | Both micro- and macrovascular disease vs. dual absence | |
| --- | --- | --- | --- | --- | --- | --- | --- | --- | --- | --- |
|  | Dual absence (n=5473) | Microvascular  Alone (n=2077) | Macrovascular  Alone (n=2427) | Both  (n=1163) | HR  (95% CI) | P | HR  (95% CI) | P | HR  (95% CI) | P |
| All-cause mortality | 823 (1.7) | 514 (3.0) | 501 (2.4) | 427 (4.9) | 1.36  (1.21 - 1.53) | <0.0001 | 1.42  (1.26 - 1.59) | <0.0001 | 1.99  (1.76 - 2.26) | <0.0001 |
| Major macrovascular events | 747 (1.6) | 400 (2.5) | 620 (3.3) | 399 (5.0) | 1.30  (1.14 - 1.47) | <0.0001 | 2.04  (1.83 - 2.28) | <0.0001 | 2.52  (2.21 - 2.88) | <0.0001 |
| Cardiovascular death | 276 (0.6) | 218 (1.3) | 271 (1.3) | 223 (2.5) | 1.68  (1.40 – 2.03) | <0.0001 | 2.30  (1.94 – 2.74) | <0.0001 | 2.95  (2.43 – 3.58) | <0.0001 |
| Myocardial infarction | 246 (0.5) | 121 (0.7) | 212 (1.1) | 143 (1.7) | 1.21  (0.96 – 1.53) | 0.10 | 1.90  (1.57 – 2.29) | <0.0001 | 2.60  (2.08 – 3.26) | <0.0001 |
| Stroke | 352 (0.7) | 161 (1.0) | 302 (1.5) | 153 (1.8) | 1.15  (0.94 – 1.39) | 0.18 | 2.20  (1.88 – 2.58) | <0.0001 | 2.20  (1.80 – 2.70) | <0.0001 |
| Major clinical microvascular events | 269 (0.6) | 243 (1.5) | 149 (0.7) | 146 (1.8) | 2.49  (2.06 – 3.00) | <0.0001 | 1.32  (1.08 – 1.62) | 0.007 | 2.97  (2.39 – 3.69) | <0.0001 |
| Retinal photocoagulation or blindness | 232 (0.5) | 195 (1.2) | 132 (0.7) | 110 (1.3) | 2.52  (2.06 – 3.09) | <0.0001 | 1.39  (1.12 – 1.73) | 0.003 | 2.96  (2.33 – 3.77) | <0.0001 |
| ESRD or renal death | 44 (0.09) | 62 (0.4) | 18 (0.09) | 44 (0.5) | 2.45  (1.60 – 3.78) | <0.0001 | 0.92  (0.53 – 1.61)- | 0.77 | 3.01  (1.90 – 4.79) | <0.0001 |

Dual absence means absence of both macrovascular and microvascular disease at baseline. HRs estimated using Cox proportional hazards regression models, adjusting for sex, age, region of origin (established market economies, Eastern Europe and Asia), BMI, duration of diabetes, HbA1c, systolic blood pressure, antihypertensive treatment, urinary albumin-creatinine ratio (normoalbuminuria, microalbuminuria and macroalbuminuria), LDL- and HDL-cholesterol, history of ever smoking, and randomized study allocations.

**Additional Table S3. Distibution of patients, event rate (per 100 person years) and hazard ratios (HRs) for outcomes during follow-up, according to the history of microvascular (including peripheral neuropathy) or macrovascular disease at baseline**

|  | History of microvascular or macrovascular disease  Number of events (event rate) | | | | Microvascular disease  alone vs. dual absence | | Macrovascular disease  alone vs. dual absence | | Both micro- and macrovascular disease vs. dual absence | |
| --- | --- | --- | --- | --- | --- | --- | --- | --- | --- | --- |
|  | Dual  absence (n=5242) | Microvascular  alone (n=2308) | Macrovascular  alone (n=2391) | Both  (n=1199) | HR  (95% CI) | P | HR  (95% CI) | P | HR  (95% CI) | P |
| All-cause mortality | 790 (1.7) | 547 (2.8) | 543 (2.7) | 385 (4.2) | 1.33  (1.18 – 1.49) | <0.0001 | 1.50  (1.34 – 1.68) | <0.0001 | 1.74  (1.53 – 1.99) | <0.0001 |
| Major macrovascular events | 759 (1.7) | 388 (2.1) | 647 (3.5) | 372 (4.4) | 1.14  (1.01 – 1.30) | 0.04 | 2.02  (1.81 – 2.25) | <0.0001 | 2.24  (1.96 – 2.56) | <0.0001 |
| Cardiovascular death | 301 (0.6) | 193 (1.0) | 282 (1.4) | 212 (2.3) | 1.26  (1.04 – 1.52) | 0.02 | 2.05  (1.73 – 2.42) | <0.0001 | 2.50  (2.06 – 3.02) | <0.0001 |
| Myocardial infarction | 236 (0.5) | 131 (0.7) | 209 (1.1) | 146 (1.7) | 1.14  (0.91 – 1.42) | 0.27 | 1.86  (1.54 – 2.25) | <0.0001 | 2.45  (1.97 – 3.06) | <0.0001 |
| Stroke | 357 (0.8) | 156 (0.8) | 330 (1.7) | 125 (1.4) | 1.10  (0.90 – 1.34) | 0.34 | 2.30  (1.97 – 2.68) | <0.0001 | 1.86  (1.50 – 2.32) | <0.0001 |
| Major clinical microvascular events | 249 (0.5) | 263 (1.5) | 157 (0.8) | 138 (1.6) | 2.37  (1.97 – 2.85) | <0.0001 | 1.45  (1.18 – 1.77) | 0.0004 | 2.54  (2.04 – 3.16) | <0.0001 |
| Retinal photocoagulation or blindness | 209 (0.5) | 218 (1.2) | 136 (0.7) | 106 (1.2) | 2.54  (2.08 – 3.09) | <0.0001 | 1.57  (1.26 – 1.95) | <0.0001 | 2.69  (2.10 – 3.43) | <0.0001 |
| ESRD or renal death | 49 (0.1) | 57 (0.3) | 24 (0.1) | 38 (0.4) | 1.65  (1.07 – 2.55) | 0.02 | 1.03  (0.63 – 1.69) | 0.91 | 2.08  (1.29 – 3.34) | 0.003 |

Dual absence means absence of both macrovascular and microvascular disease at baseline. HRs estimated using Cox proportional hazards regression models, adjusting for sex, age, region of origin (established market economies, Eastern Europe and Asia), BMI, duration of diabetes, HbA1c, systolic blood pressure, antihypertensive treatment, eGFR and its square, urinary albumin-creatinine ratio (normoalbuminuria, microalbuminuria and macroalbuminuria), LDL- and HDL-cholesterol, history of ever smoking, and randomized study allocations.

Additional Table S4. **Subdistribution hazard ratios for outcomes during follow-up according to the history of microvascular or macrovascular disease at baseline with correction for competing risk of non-renal and non-cardiovascular death**

|  | Microvascular disease  alone vs. dual absence | | Macrovascular disease  alone vs. dual absence | | Both micro- and macrovascular disease vs. dual absence | |
| --- | --- | --- | --- | --- | --- | --- |
|  | SHR  (95% CI) | P | SHR  (95% CI) | P | SHR  (95% CI) | P |
| Major macrovascular events | 1.42 (1.09 – 1.85) | 0.01 | 1.97 (1.73 – 2.24) | <0.0001 | 2.59 (1.91 – 3.50) | <0.0001 |
| Myocardial infarction | 1.62 (1.10 – 2.40) | 0.02 | 1.94 (1.60 – 2.37) | <0.0001 | 3.45 (2.30 – 5.17) | <0.0001 |
| Stroke | 1.23 (0.87 – 1.74) | 0.24 | 2.07 (1.77 - 2.43) | <0.0001 | 1.98 (1.31 – 3.01) | 0.001 |
| Major clinical microvascular events | 4.97 (3.94 – 6.28) | <0.0001 | 1.36 (1.11 – 1.65) | 0.003 | 5.97 (4.40 – 8.12) | <0.0001 |
| Retinal photocoagulation or blindness | 5.15 (4.06 – 6.53) | <0.0001 | 1.42 (1.16 – 1.74) | 0.001 | 6.52 (4.78 – 8.89) | <0.0001 |
| ESRD | 1.41 (0.48 – 4.11) | 0.53 | 0.65 (0.29 – 1.40) | 0.27 | 1.05 (0.29 – 3.82) | 0.94 |

Dual absence means absence of both macrovascular and microvascular disease at baseline. Subdistribution hazard ratios (SHR) adjusted for sex, age, region of origin (established market economies, Eastern Europe and Asia), BMI, duration of diabetes, HbA1c, systolic blood pressure, antihypertensive treatment, eGFR and its square, urinary albumin-creatinine ratio (normoalbuminuria, microalbuminuria and macroalbuminuria), LDL- and HDL-cholesterol, history of ever smoking, and randomized study allocations.
